# Effects of lime application on nitrogen and phosphorus availability in humic soils

**DOI:** 10.1038/s41598-020-65501-3

**Published:** 2020-05-25

**Authors:** N. P. Mkhonza, N. N. Buthelezi-Dube, P. Muchaonyerwa

**Affiliations:** School of Agricultural, Earth and Environmental Sciences, University of KwaZulu-Natal, Private Bag X01, Scottsville, 3209 South Africa

**Keywords:** Environmental sciences, Environmental sciences, Environmental sciences, Sustainability, Sustainability

## Abstract

There is a paucity of information on nitrogen (N) and phosphorus (P) mineralization in humic soils, which are highly weathered and have high carbon (C) (>1.8%). This study was to determine effects of liming on N and P mineralization in humic soils. Lime was applied to reduce acid saturation to 20% of the 0–10 and 10–20 cm depths of soils from Eston and Eshowe. Soils were incubated at field capacity moisture and 25 °C temperature, with destructive sampling after 0, 7, 14, 21, 28, 56, 84 and 112 days. Samples were analysed for pH, ammonium- and nitrate-N and extractable P. Phosphorus pools and soil microbial biomass C and N (SMBC and N) were analysed after 112 days only. Soil pH increased up to day 7 and decreased thereafter in Eston soil but decreased throughout the incubation in Eshowe soil. Ammonium- and nitrate-N increased with lime rate, with ammonium-N peaking after 7 and 14 days for Eston and Eshowe soils, respectively. The 0–10 cm depth had higher ammonium-N than 10–20 cm for both soils. Nitrate-N increased with corresponding decrease in ammonium-N. Extractable P decreased till day 21 and increased thereafter in Eston soil, with slight changes in Eshowe. Higher lime rate decreased Al-P, Fe-P and CBD-P and increased soluble-P, Ca-P, and SMB-C and N for both soils. The findings imply that liming humic soils increase nitrate-N and, to a lesser extent, extractable P, possibly improving productivity and exposing N to leaching.

## Introduction

Liming is commonly used to improve the productivity of acidic soils in agricultural systems. The addition of lime increases the availability of nutrients, which would otherwise be strongly limited by low soil pH^1^. An increase in soil pH, as a result of liming, increases microbial activity^2–4^, resulting in increased decomposition of resident SOM^5,6^. The decomposition could result in increased mineral N and P and losses of SOC through CO_2_ emission^7,8^. Generally, increasing soil pH to >5.5 would be optimum for the development and growth of nitrifiers^9^. High rates of nitrification have been reported when lime is applied to strongly acidic soil^10^. Zhao *et al*.^11^, Lyngstad^12^ and Yao *et al*.^13^ reported increases in nitrate-N after application of lime to forestry soils. The increase in nitrification can be explained by high microbial activity following lime application attributed to an increase in soil pH^2–4,14^. However, other results have shown liming to either have no effect^15^ or a decrease^16^ in microbial activity of highly acidic soils. These differences could be due to soil physico-chemical properties such as total C and N, C: N ratio and soil pH. The differences in microbial response could also be due to differences in lime requirements of the soils (e.g. soils with high acid saturation would require more lime) and also differences in organic matter contents. The response of microbial communities and mineralization process to liming also varies with soil type and management conditions^5^. No effects or decreased mineralization with lime application could be attributed to increased protection of organic substrates due to increase aggregates stability^17^. In addition to increased microbial activity, lime addition increases P availability in highly acidic soils^18^. The lime application increases P availability by decreasing Al^3+^ and Fe^3+^ ions and fixation on aluminium and iron oxides while over-liming to pH values up to 8.5 could result in precipitation of calcium phosphate^19^. While the role of Al-organic matter complexes in fixing P is not clearly understood, the decomposition of the organic matter may release the bound Al into the soil solution, due to liming, with the possible increase in fixation of P. This effect has not been ascertained, including in humic soils of South Africa.

Humic soils are intensely weathered and highly acidic and are characterised by high C (>1.8%) content, high buffering capacity, and good drainage, low bulk density and base status^20^. These soils are restricted to regions receiving high rainfall and cool temperatures mostly in parts of the Eastern Cape, KwaZulu-Natal and Mpumalanga in South Africa^20^. There is no evidence, in literature, of the occurrence of these soils other than in South Africa. The characteristics of these soils are relatively similar to those of Andosols but the conditions on which they are formed are different, and the organic matter concentrations in some humic soils are higher. From our preliminary experiments, some humic soils have as high as 10% organic C and such levels of organic matter could results in excessive mineralization of N with potential for leaching to ground water. Like in Andosols, formation of Al-organic matter and Fe-organic matter complexes^21^ is believed to be a major mechanism of soil organic matter (SOM) stabilisation in humic soils. These organo-mineral complexes increase the protection of SOM from microbial decomposition^22^. The effects of liming on nitrogen mineralization and P availability could be magnified in the highly productive humic soils.

A large portion of humic soils in KwaZulu-Natal is under sugarcane, where treatment with lime is used to reduce the effects of acidity. The reduction in acidity could increase microbial activity, decomposition of SOM and mineralization of nutrients, making a significant contribution to crop N and P requirements, while the release of Al bound to SOM could increase exchangeable acidity or moderate effects of lime. Depending on the concentration of mineral N from decomposition of resident SOM, crop requirements could be achieved, with possible excess N contaminating ground water. The good drainage of humic soils^20^ increases the possibilities of any excess nitrates to be leached. This effect could be particularly important with increase in demand for arable land which could result in humic soils under forestry and grassland being converted to cultivation with consequent inevitable need for lime. There is a need to understand the decomposition of SOM in humic soils especially those under forestry, which could be converted to arable land. Considering the high C content stored within these soils^20^, which could be altered following liming, there is a need to understand the effects of lime application on pH, concentrations mineral N and P in disturbed humic soils under forestry. The objective of the study was to determine the effects of liming on N and P availability from humic soils during incubation.

## Results

### Physicochemical properties of humic soils before liming

Both soils had sandy clay loam texture with over 50% sand (Table 1). The soil from Eston had 9.89% C and 0.73% N (C: N = 14:1) in the 0–10 cm depth while that from Eshowe had 6.04% C and 0.38% N (C: N = 16:1). The Eston soil had >80% acid saturation for both depths while the soil from Eshowe had 3% in the 0–10 cm and 28% in the 10–20 cm depth (Table 1). The soil from Eston had higher extractable P and exchangeable K, with lower exchangeable Ca than that from Eshowe.

**Table 1:** Selected physico-chemical characteristics of the forestry humic soils used.

Property	Eston		Eshowe		LSD
Depth (cm)	0–10	10–20	0–10	10–20	
pH_(KCl)_	3.95	3.96	5.16	4.35	0.24
pH_(H2O)_	4.66	4.79	5.91	4.62	0.50
Carbon (%)	9.89	4.26	6.04	3.80	1.77
Nitrogen (%)	0.73	0.21	0.38	0.18	0.18
C: N	14: 1	20: 1	16: 1	21: 1	
Sand (%)	57.7	61	53. 7	53.7	23.9
Silt (%)	11	10	16.7	14.7	9.88
Clay (%)	30.7	29	30	33.3	15.1
Extractable P (mg/kg)	9.87	7.29	5.89	6.11	0.79
Exchangeable K (cmol_c_/kg)	0.68	0.29	0.09	0.18	0.04
Exchangeable Ca (cmol_c_/kg)	0.53	0.32	3.11	1.45	0.70
Exchangeable Mg (cmol_c_/kg)	3.50	0.24	2.96	1.40	0.18
Exchangeable Na (cmol_c_/kg)	1.75	1.90	1.82	1.76	0.08
Exchangeable Acidity (cmol_c_/kg)	4.28	3.89	0.20	1.2	0.08
Acid saturation (%)	81	83	3	28	

### Effect of liming on soil properties

#### Soil pH

**Figure 1:**
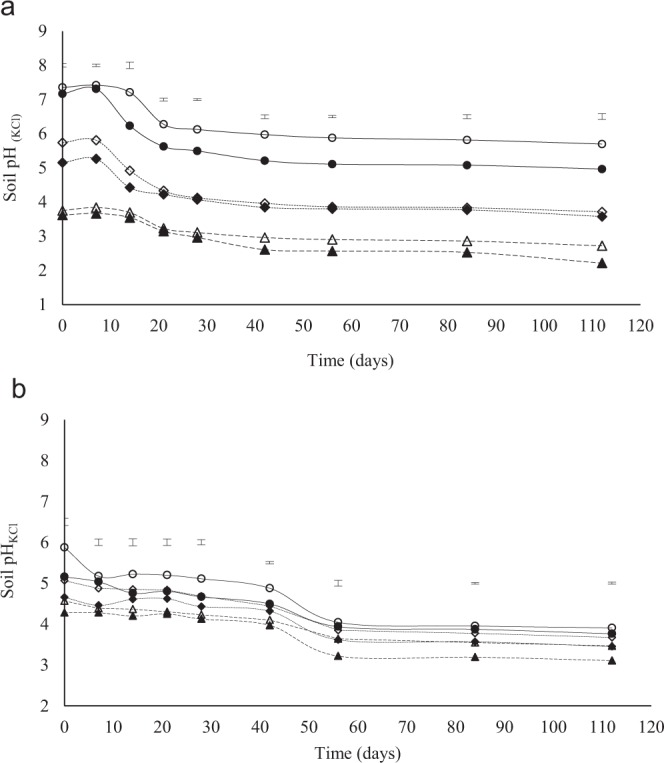
Changes in **s**oil pH_(1M KCl)_ from different application rates of lime at different depth (0–10 and 10–20 cm). The letters A and B indicates Eston and Eshowe soil, respectively. Lime was applied at a rates of 0, 12 and 36 t ha^−1^ for Eston and 0, 1.5 and 4.5 t ha^−1^ for Eshowe soil. Vertical error bars indicates LSD (p < 0.05). The no fill and fill markers represent 0–10 and 10–20 cm depth, respectively. Symbols ◊ 12 t ha^−1^ (0–10 cm); ♦ 12 t ha^−1^ (10–20 cm); ∆ control (0–10 cm); ▲ control (10–20 cm); o 36 t ha^−1^ (0–10 cm); • 36 t ha^−1^ (10–20 cm).

There were significant differences in pH among all lime treatments and depth, for each sampling period, throughout the incubation study except that the difference between the two depths for the recommended lime rate was only significant up to day14 in the Eston soil (Fig. 1A). A rapid decrease was observed between days 7 and 28 (Fig. 1A), followed by a slower decline until day 112 for all treatments and at both depths. The higher lime application rate had higher pH compared to the recommended rates and the control with the 0–10 cm depth, in all treatments, being higher in pH than the 10–20 cm (Fig. 1A). There was a slow decline in pH in the Eshowe soil up to day 42, followed by a sharp decline between days 42–56, and no further change was observed till day 112 (Fig. 1B).

### Ammonium-N

**Figure 2:**
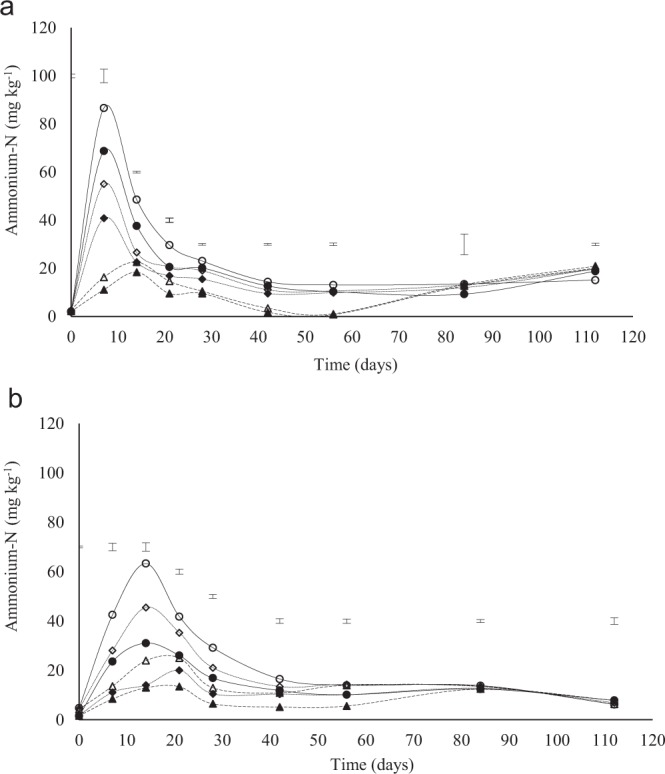
Ammonium-N concentration at different application rates of lime at different depth (0–10 and 10–20 cm). Lime was applied at a rates of 0, 12 and 36 t ha^−1^ for Eston and 0, 1.5 and 4.5 t ha^−1^ for Eshowe soil. The letters A and B indicates Eston and Eshowe soil, respectively. Vertical error bars indicates LSD (p < 0.05). The no fill and fill markers represent 0–10 and 10–20 cm depth, respectively. Symbols ◊ 12 t ha^−1^ (0–10 cm); ♦ 12 t ha^−1^ (10–20 cm); ∆ control (0–10 cm); ▲ control (10–20 cm); o 36 t ha^−1^ (0–10 cm); • 36 t ha^−1^ (10–20 cm).

There was a sharp increase in ammonium-N concentration within the first 7 days in the Eston soil (Fig. 2A). Significant differences in ammonium-N concentrations, among all treatments, were observed up to day 56 (Fig. 2A). There was a higher ammonium-N concentration in amended soils from the 0–10 cm depth compared to 10–20 cm (Fig. 2A). Between days 56–112 (Fig. 2A) ammonium-N concentration in the control increased. Higher ammonium-N concentration occurred at higher application rates of lime for both depths in the Eston soil (Figure 3.2A) and for 0–10 cm depth in the Eshowe soil (Fig. 2B). At the recommended rate, the Eston soil had a maximum ammonium N concentration of 55 mg kg^−1^. The same trend in ammonium-N was observed for Eshowe with a maximum at day 14. Between days 56–84 ammonium-N concentrations in the control increased and then decreased from day 84–112 in all treatments (Fig. 2B). The maximum ammonium-N concentration at the recommended lime rate in Eshowe soil was 45 mg kg^−1^.

### Nitrate-N

**Figure 3:**
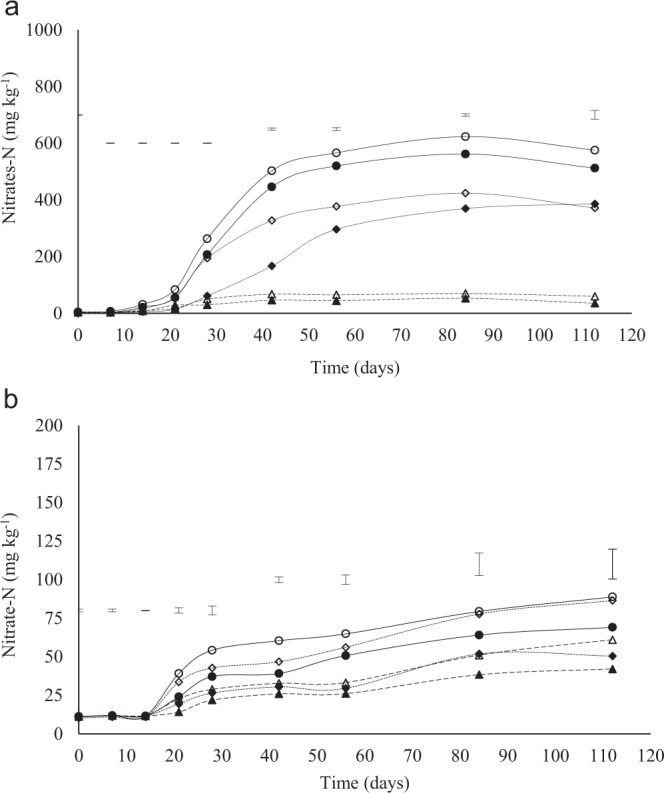
Concentration of nitrate-N at different application rates of lime at different depth (0–10 and 10–20 cm). Lime was applied at a rates of 0, 12 and 36 t ha^−1^ for Eston and 0, 1.5 and 4.5 t ha^−1^ for Eshowe soil. The letters A and B indicates Eston and Eshowe soil, respectively. Vertical error bars indicates LSD (p < 0.05). The no fill and fill markers represent 0–10 and 10–20 cm depth, respectively. Symbols ◊ 12 t ha^−1^ (0–10 cm); ♦ 12 t ha^−1^ (10–20 cm); ∆ control (0–10 cm); ▲ control (10–20 cm); o 36 t ha^−1^ (0–10 cm); • 36 t ha^−1^ (10–20 cm).

Nitrate-N concentrations were only significantly different from 14 days of incubation for the Eston soil (Fig. 3A). The nitrate-N rapidly increased from day 14–56, and thereafter, a slower increase was observed in all treatments except for the control, where minimal change was observed, throughout the incubation period (Fig. 3A). Higher nitrate-N concentration was observed at higher lime application rates for Eston soil (Fig. 3A). Higher nitrate-N concentration occurred in the 0–10 cm depth within each lime treatment compared to 10–20 cm depth (Figure. 3.3A). Treatment and depth differences in soil nitrate-N were significant from 21 days in the Eshowe soil (Fig. 3B). Higher nitrate-N concentration occurred in the 0–10 cm depth of the Eshowe soil amended with lime (Fig. 3B). The Eston soil had nitrate N as high as >400 mg kg^−1^ at the recommended rate and >600 mg kg^−1^ at triple the recommended lime rate. The highest nitrate-N concentration in the Eshowe soil was less than 100 mg kg^−1^, even at triple the recommended lime rate.

### Extractable P

**Figure 4:**
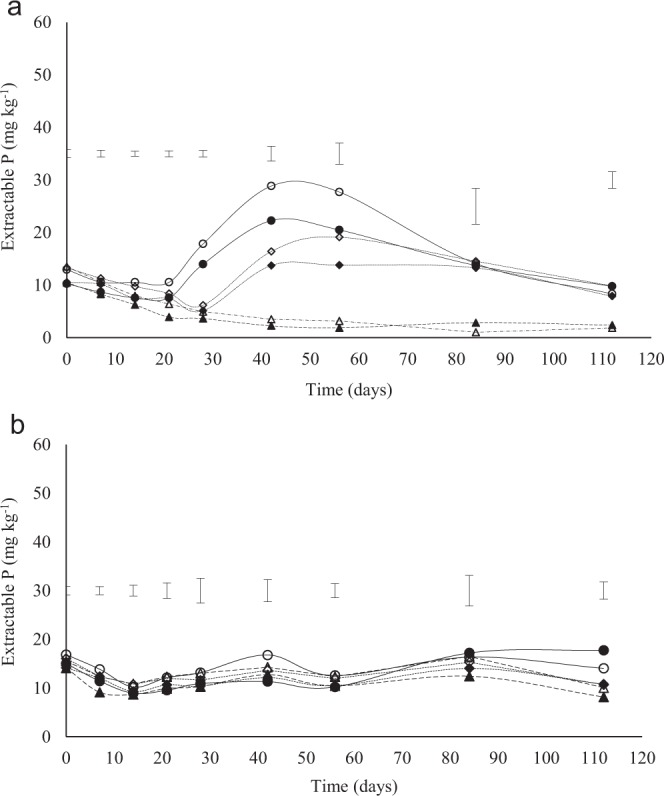
Extractable P concentrations at different application rates of lime at different depth (0–10 and 10–20 cm). Lime was applied at a rates of 0, 12 and 36 t ha^−1^ for Eston and 0, 1.5 and 4.5 t ha^−1^ for Eshowe soil. The no fill and fill markers represent 0–10 and 10–20 cm depth, respectively. Symbols ◊ 12 t ha^−1^ (0–10 cm); ♦ 12 t ha^−1^ (10–20 cm); ∆ control (0–10 cm); ▲ control (10–20 cm); o 36 t ha^−1^ (0–10 cm); • 36 t ha^−1^ (10–20 cm).

Extractable P decreased within the first 21 days of incubation of the soil from Eston (Fig. 4A). From day 21 to 42 there was an increase in extractable P in all treatments except the control (Fig. 4A). More extractable P occurred in the 0–10 compared to 10–20 cm depth (Fig. 4A). A decrease of extractable P concentration in soils amended at triple the lime rate was observed from day 56 to 84 where the P concentration was not significantly different except for the control, which had lower concentration up to 112 days (Fig. 4A). There was a decline in extractable P in all treatments in the soil from Eshowe up to day 14, thereafter there was a slight increase with no significant change (Fig. 4B). There was no significant difference in extractable P within treatment and depth from day 0–84 (Fig. 4B). The significant difference occurred on day 112 where the triple application rate had a significantly higher concentration than the recommended rates and control for both depths (Fig. 4B). The Eston soil had a maximum extractable P of about 20 mg kg^−1^ at the recommended rate, and about 30 mg kg^−1^, at triple the lime rate, while for the Eshowe soil the highest was about 17 mg kg^−1^.

### Phosphorus fractionation

#### Eston Soil

**Figure 5:**
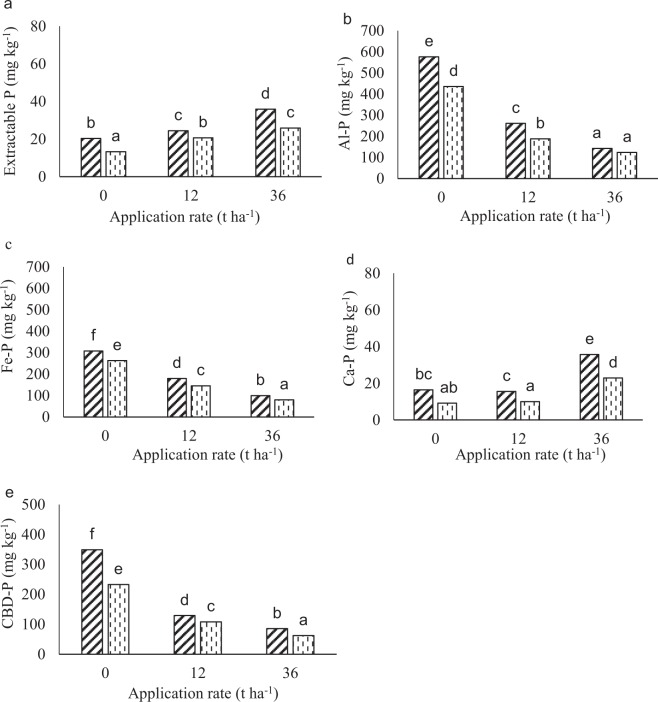
Phosphorus fractions at different application rates of lime at 112 days of incubation in the Eston soil. Figure A, B, C, D and E indicates soluble-P, Al-P, Fe-P, Ca-P and CBD-P, respectively. Lime was applied at a rates of 0, 12 and 36 t ha^−1^ at different depth of 0–10 and 10–20 cm. Diagonally striped bars and vertically dashed bars represent 0–10 and 10–20 cm depth, respectively. Bars with same letters are not significantly different.

There were significant differences in soluble-P between depths and among treatments. Soluble P was higher in the 0–10 than the 10–20 cm depth for all lime treatments. The soluble P concentration also increased with an increase in lime rate (Fig. 5A). The Al-P, Fe-P and CBD-P decreased with an increase in lime application rates (Fig. 5B,C,E). The 0–10 cm depth had higher Al-P, Fe-P and CBD-P than the 10–20 cm depth for all lime rates except at 36 t ha^−1^ where there was no difference in Al-P (Fig. 5B). The Ca-P fraction was generally lower than the other fractions in all the treatments. The 0–10 cm depth had higher Ca-P than the 10–20 cm depth for the lime rates except in the control (0 t ha^−1^), where there was no difference (Fig. 5D). The 36 t ha^−1^ lime had higher Ca-P than the other rates, irrespective of depth.

#### Eshowe soil

**Figure 6:**
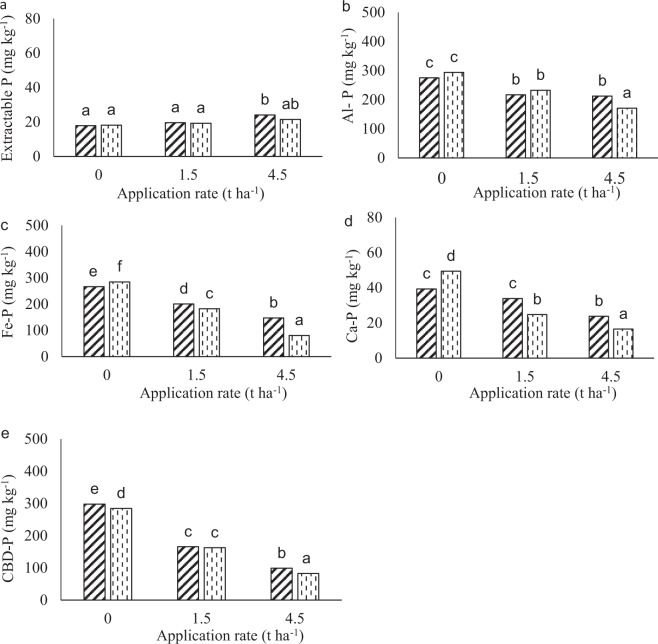
Eshowe soil P fractions at different application rates of lime at 112 days of incubation. Lime was applied at a rates of 0, 1.5 and 4.5 t ha^−1^ at different depth of 0–10 and 10–20 cm. Figure A, B, C, D and E indicate soluble-P, Al-P, Fe-P, Ca-P and CBD-P, respectively. Diagonally triped bars and vertically dashed bars represent 0–10 and 10–20 cm depth, respectively. Bars with same letters are not significantly different.

There was no significant difference in soluble P treatments except for the 4.5 t ha^−1^ in the 0–10 cm depth (Fig. 6A). There was a significant decrease in Al-P and Fe-P with the increase in lime rate (Fig. 6B.C), except that there were no differences in Al-P between the 1.5 and 4.5 t ha^−1^ in the 0–10 cm depth. Soil depth did not affect Al-P except at 4.5 t ha^−1^ rate, where the 0–10 cm depth had higher concentrations than the 10–20 cm depth. The Fe-P (Fig. 6C) and Ca-P (Fig. 6D) concentrations declined with soil depth except in the control where the 10–20 cm depth had a higher concentration. The CBD-P also decreased with the increase in application rates of lime and soil depth (Fig. 6E).

### Soil microbial biomass carbon (SMB-C) and nitrogen (SMB-N)

**Figure 7:**
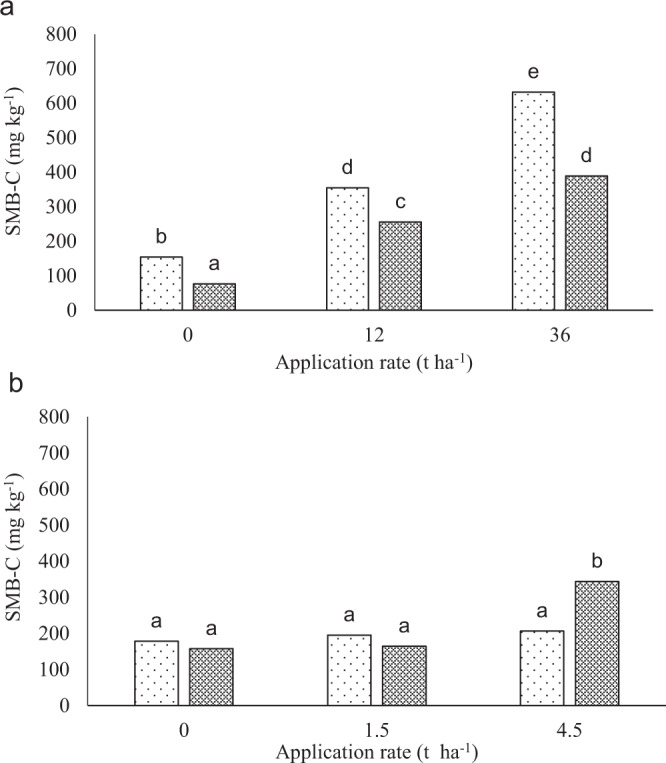
Soil microbial biomass carbon at different application rates of lime at 112 days of incubation. Figure A and B indicates Eston and Eshowe soil, respectively. Lime was applied at a rates of 0, 12 and 36 t ha^−1^ and 0, 1.5 and 4.5 t ha^−1^ at different depth of 0–10 and 10–20 cm for Eston and Eshowe, respectively. The white dotted and black hatched bars represent 0–10 and 10–20 cm depth, respectively. Bars with same letters are not significantly different.

**Figure 8:**
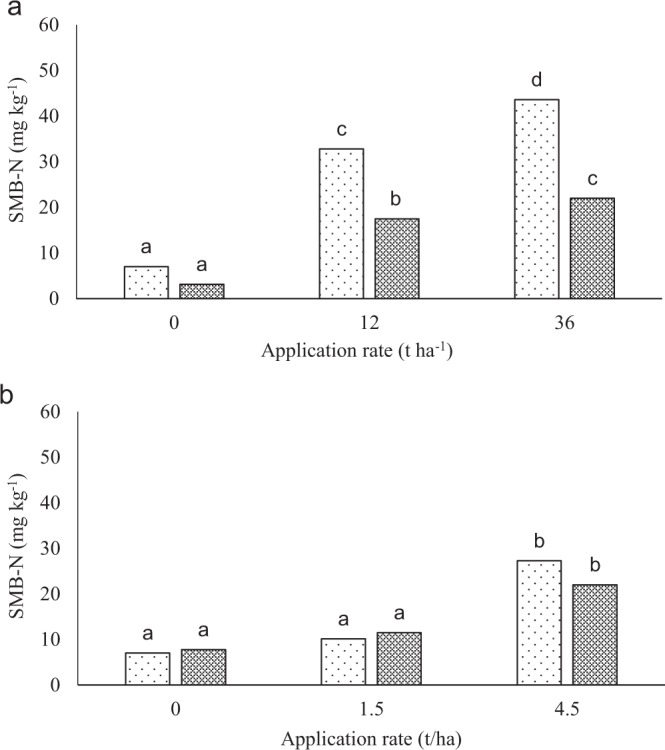
Soil microbial biomass nitrogen at different application rates of lime at 112 days of incubation. Figure A and B indicates Eston and Eshowe soil, respectively. Lime was applied at a rates of 0, 12 and 36 t ha^−1^ and 0, 1.5 and 4.5 t ha^−1^ at different depth of 0–10 and 10–20 cm for Eston and Eshowe, respectively. The white dotted bars and black hatched bars represents 0–10 and 10–20 cm depth, respectively. Bars with same letters are not significantly different.

There were significant differences in SMB-C (Fig. 7A) among the treatments and between the depths after 112 days of incubation in the Eston soil. The SMB-C increased with an increase in lime rate at both depths (Fig. 7A). The 0–10 cm depth had higher SMB-C than the 10–20 cm depth. In the Eshowe soil, only the 4.5 t ha^−1^ lime rate had higher SMB-C in the 10–20 cm depth (Fig. 7B). There were significant differences in SMB-N (Fig. 8A) among the treatments and between the depths after 112 days of incubation in the Eston soil. The 0–10 cm depth had higher SMB-N than the 10–20 cm depth, except in the control where there were no differences between the depths. In the Eshowe soil, only the 4.5 t ha^−1^ lime rate had higher SMB-N at both depths (Fig. 8B) than all the other treatment combinations.

## Discussion

The increase in pH with an increase in lime rate (Fig. 1A) was due to an increase in hydroxide ions following lime application^23^. The increase in pH supported greater microbial activity^2^, resulting in greater decomposition of organic matter^6^, resulting in the increase in ammonium-N concentrations in the 0–7 and 0–14 days periods for the soils from Eston and Eshowe, respectively (Fig. 2A,B). An increase in pH upon the addition of lime removes acidity as a constraint, increasing microbial activities resulting in increased mineralization of N^3^. Anderson^24^; Chagnon *et al*.^25^ reported that an increase in soil pH after liming results in a significant increase of SMB-C. Similar results were reported by several authors^14,26–28^. The increase in SMB-C coincided with the increase in nitrate-N (Fig. 3A) and with application rate. The decrease in pH of Eshowe soil could be due to decomposition of SOM releasing humic acids initially and Al^19^ and low level of lime applied (Fig. 1B) while lower ammonium-N concentrations was also available (Fig. 2B), than from the Eston soil (Fig. 2A), as SOM decomposed.

The highest concentration of ammonium-N at day 7 for the Eston (Fig. 2A) and 14 for the Eshowe soil (Fig. 2B) suggest that upon removal of pH constraints rapid decomposition of SOM and N mineralization occurred releasing ammonium-N. Baggs *et al*.^29^ reported similar results, where ammonium-N was higher after the addition of lime due to increased mineralization rates. The decrease in pH between day 7 and day 28 (Fig. 1A) was attributed to nitrification, which released H^+^ ions. The sharp decrease in ammonium-N from day 7 in the Eston soil (Fig. 2A) and 14 for the Eshowe soil (Fig. 2B), corresponds with an increase in nitrate-N concentration (Fig. 3A,B). These findings are coherent with Fuentes *et al*.^9^ who reported an increase in nitrate-N with increasing lime application rate. This decrease in soil pH, while lime is applied, could also be explained by aluminium released from Al-organic matter complexes during decomposition of SOM, increasing exchangeable acidity. The nitrate-N concentrations in the limed Eston soil could be more than required by crops, and could possibly leach resulting in groundwater contamination. At triple the recommended lime rate, the Eston soil had a maximum nitrate-N concentration of 623 mg N kg^−1^ soil, which is extremely high for crop uptake^30^. For example, crops like maize require 220 kg N ha^−1^ to produce 10 t ha^−1^ ^31^ while spinach requires 100 kg N ha^−1^ for optimum yields^30^. Conversion of humic soils from wattle forestry to arable crops, with lime applied at recommended rates, could possibly result in a concentration of 787 kg N ha^−1^ (mineral-N) without any addition of inorganic fertiliser. Such excessive mineral-N concentrations as a result of liming could be lost as NO_3_-N. While lime makes N more available for crops, excessive concentrations poses risks to the environment. Although the concentrations were extremely high under conditions of continuous optimum temperature and moisture during incubation, the levels could be lower under field conditions, where wetting and drying occur and where temperatures fluctuate. Potential leaching of nitrate-N from cultivated humic soils and its concentration in groundwater needs to be evaluated since these acidic soils are usually limed when used for cropping. Generally, most crops require between ~20–400 kg N ha^−1^ for optimum yields except for some pastures, nuts and fruits which require very low N^30^. The differences in nitrate-N between the two soils used could be explained by differences in lime requirements due to differences in acid saturation of >80% and <30% for Eston and Eshowe soil, respectively (Table 1). The soil from Eston also had higher organic C and N than that from Eshowe, further explaining the differences in the magnitude of nitrate-N concentrations. Furthermore, the Eston soils had pH >5.5 following lime application at triple rate while Eshowe had pH <5.5 which inhibits nitrifiers^9^. Higher ammonium and nitrate-N at 0–10 cm depth could be explained by the higher total C and N contents (Table 1) at a shallower depth, which acts as energy and nutrient source for microbes. These findings are also evidenced by the lower C: N ratio (Table 1) at depth 0–10 than 10–20 cm, supporting the higher mineralization rates that occurred at the 0–10 cm depth. The increase in ammonium-N concentration from day 56–112 (Fig. 2A) and day 56–84 (Fig. 2B) in the control could possibly be due to the death of microbes releasing N as ammonium-N back into the soil solution.

Acidic soils have high P fixation capacity due to high Al and Fe decreasing its availability^32^. The decrease in extractable P from day 0–28 (Fig. 4A,B) could be due to its immobilization by soil microorganisms and its fixation as Al- and Fe-phosphates and on Al and Fe oxide (Figs. 5B,C and 6B,C) in the highly weathered and acidic soil conditions. This is so, because addition of lime increases microbial communities^2^, hence, increasing decomposition of SOM and release of Fe and Al^19^. The increase in extractable P between days 21 and 56 for the limed treatments, in the Eston soil, with 80% initial acid saturation, shows that microbial immobilization contributed to the decline in the first 21 days, and then released the P when the microbes died. Furthermore, the lowest observed extractable P coincided with highest Al and Fe bound P. Significant P fixation recorded for the control (decrease to almost zero after 28 days) suggests a concern of limited available P and its implications for the productivity of humic soils. After application of lime microbial activities initially increased and could have led to immobilization of extractable P. The decrease in Al-P and Fe-P could be due to their precipitation as insoluble Al(OH)_3_ and Fe(OH)_3_ after increased addition of liming material^23^. In addition, Al and Fe oxides become more negatively charged with an increase in pH contributing to an increase in available P^33,34^. The decrease in the fractions with an increase in lime rate could be due to soil pH >4 at day 112 in triple application rate for Eston soil (Fig. 1A) as maximum adsorption of P as Al-P or Fe-P occurs at pH <4^18^. As a result, Fe and Al bound P decreased as pH increased. Even at triple the recommended lime rate the Al-P, Fe-P and CBD-P were still >80 mg kg^−1^, suggesting that such high rates did not eliminate the P fixation capacity of these soils. This could be because the recommendation rate did not account for Al from Al-organic matter complexes, which are released when the organic matter is decomposed and is hydrolysed to cause acidity. This corresponds with the high extractable P available at high application rates of lime after 28 days (Fig. 4A). Low P was fixed on calcium because of low calcium content in highly weathered soils^20^. The increase in Ca-P with application rates of lime could be explained by an increase in calcium concentration after the addition of lime (calcium carbonate), especially at high pH. These findings are similar to Kiflu *et al*.^35^ observation where Ca-P increased with lime application.

## Conclusion and recommendation

Application of lime to humic soils increases mineral-N (ammonium- and nitrate-N) and to a lesser extent, extractable P. Liming the strongly acidic humic soils at a recommended rate or higher released excessive amounts of nitrate-N when incubated under optimum conditions of temperature and moisture. Higher lime rates increased soluble P, microbial biomass C and N, and reduced P associated with Al and Fe and their oxyhydroxides. Even at triple the recommended lime rate, Al-P, Fe-P and CBD-P still occurred at >80 mg kg^−1^. The increase in mineral-N and P could be sufficient to increase crop productivity, without the addition of fertiliser, at least in the short term. High mineral N as a result of liming could be associated with mineralization of C resulting in high CO_2_ emissions and a decline in soil C in the long-term. Future work must be done under field conditions with up to double the recommended lime rates. Also, future research needs to be done on CO_2_ emission and leaching of nitrates from humic soils after lime application, under field conditions, where temperature and moisture fluctuate, considering the possibilities of conversion of the forestry and grasslands on humic soils to arable land for crop production.

## Methods and materials

The incubation study was conducted under laboratory conditions at the University of KwaZulu-Natal, Pietermaritzburg campus (29° 37′ 32.9″ Latitude; 30° 24′ 18.8″ Longitude) in KwaZulu-Natal Province of South Africa.

### Soils

Two humic soils, collected from Eshowe (−25° 53′ 18″ Latitude; 31° 26′ 10″ Longitude) and Eston (−29° 55′ 41″ Latitude; 30° 27′ 7″ Longitude) farms, were used in the study. The soils were both underlain by sandstone as a parent material. The soil from Eston was under wattle (*Acacia pycnantha*) while at Eshowe it was under natural forest. Both soils were both classified as Magwa soil form with humic A horizon overlying yellow-brown apedal B horizon over unspecified material^36^, which was translated to Ferralsols according to the world classification system^37^. These soils are highly weathered, well-drained, acidic soils with low base status and high organic carbon content >1.8%^20^. Both soils had sandy clay loam texture. The soils were sampled from the 0–10 and 10–20 cm depth, using micropits, air-dried and sieved (<2 mm) before analysis.

### Soil analysis

The soils were analysed for pH in water and 1 M KCl, extractable phosphate, exchangeable acidity and bases, total carbon and nitrogen, particle size distribution and bases, using methods describe by Non-Affiliated Soil Analysis Work Committee^38^. Soil pH was determined with 10 g of air dried soil suspended in 25 ml of 1 M KCl or water. Phosphorus was extracted by the AMBIC-2 method as described by Non-Affiliated Soil Analysis Work Committee^38^. For each sample, soil (2.5 g) was transferred into individual centrifuge tubes and then 25 mL of Ammonium Bicarbonate (AMBIC-2) solution added. Extractable P was read on a UV/VIS spectrophotometer using the molybdenum-blue method^39^. Total C and N were measured using the LECO Trumac CNS Autoanalyser^40^. For this, the soil was grinded and allowed to pass through 250 µm sieve before analysis. A sample of 0.2 g was mounted into crucibles and subjected to temperature of 1350 °C furnace temperature for about 6 minutes for each sample. Exchangeable bases (Ca^2+^, Mg^2+^, K^+^ and Na^+^) were determined using ammonium acetate (pH 7) extraction method followed by quantification with atomic adsorption spectrophotometry (Varian 2600). Exchangeable acidity was determined by extraction using 1 M KCl, followed by titration with 0.1 M NaOH with phenolphthalein indicator, as described by Jones^41^. Particle size distribution was determined in bulk samples (<2 mm) from the 0–10 and 10–20 cm depth using the hydrometer method as modified Gee and Or^42^. Thereafter, textural class was determined using the textural triangle^36^.

### Incubation Experiment

The experiment was a 2 ×3 factorial in a completely randomised design for each soil. The factors were soil depth (2 levels) and lime rate (3 levels). The treatment combinations was replicated three times. The liming treatments were (i) no lime, (ii) lime added at the recommended rate and (iii) lime at triple the recommended rate. The lime (CaCO_3_) required to reduce acid saturation of the two soils to 20% (recommended rate) was determined and calculated (Equation 1) according to Manson *et al*.^30^. One hundred grams of soil were placed in plastic container and the lime treatments added, and moistened to 100% water holding capacity. The containers were loosely caped, to allow for gas exchange. The triple recommended rate was used to determine effects of an extreme case scenario. The incubation experiment was conducted in constant temperature room at 25 °C for 112 days, with sampling at 0, 7, 14, 21, 28, 42, 56, 84, 112 days. Moisture correction was done after determining weight loss.

**Lime Requirement (t ha**^−**1**^**)** = [“Exch. Acidity” - (“Total cations” x PAS/100)] x FEquation 1.

Where:

PAS is “the permissible acid saturation for the crop in question. It is the maximum acid saturation which would allow optimum crop growth, and is used in the calculation of the lime requirement”. For this study the PAS was 20% acid tolerance. F is “a factor indicating the amount of lime required to neutralize 1 cmol_c_/L of exchangeable acidity”.

Lime was applied at the rate of 0, 12 and 36 t ha^−1^ for Eston soil and 0, 1.5 and 4.5 t ha^−1^ for Eshowe soils. The recommended lime rates were 12 and 1.5 t ha^−1^ for Eston and Eshowe soils, respectively. Calcitic lime (CaCO_3_) with calcium carbonates equivalent of 95% and fineness of 2500 µm, was used.

### Determination of Ammonium- and Nitrate-N, Extractable P and Soil Microbial Biomass Carbon and Nitrogen

Ammonium- and nitrate-N were extracted with 2 M KCl and analysed using the Thermo Scientific Gallery Discrete Auto-analyser using the automate calometric hydrazine reduction^43^. For extraction of ammonium-N and nitrate-N, 2 g soil and 20 ml of 2 M KCl were used. Extractable phosphate was analysed using the AMBIC-2 method as described by Non-Affiliated Soil Analysis Work Committee^38^. Soil microbial biomass carbon and nitrogen (SMB-C and N) were determined only for the final day of incubation using the fumigation-extraction method^44^. For this, 20 g of the incubated samples was fumigated with 50 ml ethanol free chloroform in a desiccator for 24 hours at 25 °C. For extraction 0.5 M K_2_SO_4_ was used to extract SMB-C, and the same extracts were used for SMB-N determination.

### Determination of soil P fractions

Soil P fractionation was analysed as described for non-calcareous soils by Zhang and Kovar^45^, only for the last day of incubation (day 112). Soluble and loosely bound P fraction was extracted using 50 ml of 1 M ammonium chloride (1 M NH_4_Cl) for 1.0 g soil sample. The soil residue was extracted with 50 ml of 0.5 M ammonium fluoride (0.5 M NH_4_F) to determine P bound as aluminium phosphate. Phosphorus bound as Fe phosphate was extracted from the same soil residue using 50 ml of 0.1 M sodium hydroxide (NaOH). For CBD-P extraction, 40 ml of 0.3 M sodium citrate dihydrate (0.3 M Na_3_C_3_H_6_O_7_) and 5 ml of 1 M sodium bicarbonate (1 M NaHCO_3_) were added to the soil residue and heated for 15 min in a water bath at 85 °C. Thereafter, 1 g of sodium dithionate (Na_2_S_2_O_2_) was added, followed by rapid stirring, and the suspension was then heated again for 15 minutes. The extracts were then filtered using Whatman No.1 filter paper and the soil residue was washed twice using 25 ml of saturated sodium chloride. These washings were combined with each extract. Calcium bound phosphorus was extracted by treating the residue with 0.25 M sulphuric acid (0.25 M H_2_SO_4_), shaken for one hour and centrifuged at 4000 rpm for 10 minutes. Phosphorus concentration in various solutions was determined using phosphor-molybedate method^26^.

### Statistical analysis

Statistically analysis was done using repeated measures analysis of variance (ANOVA) which accounted for changes in soil pH, ammonium- and nitrate-N and extractable P. Followed by Least Significant Difference (LSD) using a two way ANOVA for lime × depth for each day. For soil P fractions and SMBC and N, ANOVA as with lime × depth interaction was used. Multiple comparison was done using Turkey at p > 0.05 to determine differences within treatments for SMBC and N.

## Data Availability

The datasets used for graphs and table are available from the corresponding author upon request.
